# Sexual Habits and Sexual Dysfunctions in a Sample of Patients with Psychotic Disorders Compared to a Group of Healthy Adults

**DOI:** 10.3390/jcm11030505

**Published:** 2022-01-19

**Authors:** Benedetta Barchielli, Tommaso Accinni, Stefano Ferracuti, Luca Carlone, Federica Petrini, Massimo Biondi, Massimo Pasquini

**Affiliations:** 1 Department of Dynamic and Clinical Psychology, and Health Studies, Sapienza University of Rome, Via degli Apuli 1, 00185 Rome, Italy; benedetta.barchielli@uniroma1.it; 2 Department of Human Neurosciences, Sapienza University of Rome, Viale dell’Università 30, 00185 Rome, Italy; stefano.ferracuti@uniroma1.it (S.F.); luca.carlone@uniroma1.it (L.C.); federica.petrini@uniroma1.it (F.P.); massimo.biondi@uniroma1.it (M.B.); massimo.pasquini@uniroma1.it (M.P.)

**Keywords:** psychosis, sexual dysfunctions, sexual life, sexual habits, sexuality

## Abstract

Background: There is a growing body of literature on the association between psychosis and sexual dysfunction. However, most studies have focused on sexual dysfunction and have not investigated the sexual lives of patients with psychosis across a broader range. Material and Methods: Consecutive patients with a diagnosis of acute psychosis or schizophrenia were recruited to the study after obtaining informed consent (*n* = 46). In addition, healthy control subjects were recruited (*n* = 52). Sociodemographic and clinical data, psychopathology, and sexual functioning were assessed. Independent sample *t*-test to determine group differences was obtained. Results: In both the male and female groups, there are significant differences between psychotic individuals and healthy controls in several areas of their sexual functioning: the control group seemed to better perceive *Couple sexuality*, *Self-eroticism*, and overall appeared to have a higher *Quality of sexual life*; on the other hand, the group of patients with psychosis displayed higher scores in *Sexual dysfunction*. Conclusions: A poor sexual quality of life may be found in patients with psychotic disorders. Assessment of sexual function in these patients is necessary to identify and manage issues and provide support and help to patients in this important area of life.

## 1. Introduction

A satisfactory sex life presupposes the ability to appropriately interact with others, acceptance of one’s sexual orientation, a certain level of self-confidence, and a properly functioning sexual physiology [1]. It is usually considered that there is a correlation between mental health disorders and sexual functioning, with positive associations between sexual dysfunction and severity of psychopathology [2].

The association between sexual dysfunction and mental health have been investigated in regard to depression [3], post-traumatic stress disorder [4], obsessive-compulsive disorder [5,6], eating disorders [7] and bipolar disorder [8]. Östman & Björkman’s [9] using a semi-structured interview, found that patient suffering from psychosis experience a failure to achieve sexual satisfaction. Some patients reported an inability to achieve orgasm while others stated that they did not feel anything, neither desire nor satisfaction, whether aroused or not. In the interviews some described themselves as ‘no longer sexual beings’ and others had lost interest in masturbation. These patients reported the absence of sexual desire in addition to specific sexual dysfunctions such as erection dysfunction, anorgasmy, and overall sexual discontent.

Besides, it has been observed that sexual fantasies in people with mental health problems do not differ from the general population. Colòn Vilar’s [10] reports that intimate fantasies are the most common, followed by exploratory, impersonal and sadomasochistic fantasies, as in the control sample. This finding suggests that, also for patients with psychosis, most fantasies are pro-social and denote the need for dyadic involvement. 

Considering that some sexual dysfunctions may be related to specific pharmacological effects of psychotropic drugs [11], a study investigated a sample of people with a first-episode psychosis (FEP) and ‘drug-free’ but with a high prevalence of sexual dysfunction, set at around 40%. Results showed that in people with FEP, the most frequent sexual dysfunctions were reduced libido and hypoactive sexual desire disorder for both genders; in particular, for men ejaculatory alterations and erectile dysfunction were the most frequent sexual deficits while for women hypo lubrification and anorgasmia were the most represented. It is important to note that most of the above studies have focused on the assessment of sexual dysfunctions in psychotic patients, addressing only rarely patients’ sexual life and sexual experience related to their own identity [12,13]: indeed, in the clinical practice of mental disorders, the issue of sexuality usually has been avoided by professionals, only occasionally taking part in rehabilitation programs for patients [14].

Therefore, it becomes increasingly clear how important it is to investigate psychological and social aspects of sexuality in patients with severe mental disorders aiming at describing this issue from a biopsychosocial perspective. Indeed, evidence is accumulating that sexuality seems significantly conditioned by social, psychological, and behavioral factors that would have to be considered for a reliable assessment of sexual dysfunctions in mental health [1,15].

The purpose of this study was to compare sexual functioning between patients with psychotic disorder and healthy adult controls.

## 2. Methods

### 2.1. Participants

Sample consisted of forty-six (46) patients with a psychotic disorder (PP) and a control (NC) of 52 healthy adults. The PP group was recruited at the Department of Human Neurosciences of Sapienza University of Rome. They all were consecutively enrolled in the Psychiatric Intensive Care Unit (PICU) and in the Outpatient Clinic for Psychosis of Policlinico Umberto I Hospital in Rome from 1 January 2018 and 31 January 2019. Inclusion criteria for people with psychosis were: (1) Patients with diagnoses of schizophrenia, schizoaffective disorder, schizophrenia spectrum disorder and other psychotic disorders (without specification and with other specification) according to DSM-5 criteria; (2) Psychotic disorder not associated with other organic or psychopathological conditions; (3) The referring psychiatrist assessed patients’ illness severity and ability to undergo the protocol assessment of the study design, ensuring the homogeneity of the sample.

Exclusion criteria for people with psychosis included: (1) age < 18; (2) significant neurological disease; (3) significant systemic illnesses or unstable medical conditions that could contribute to impaired cognition; (4) a Mini Mental State Examination (MMSE) >24. 

The NC group consisted of volunteer individuals who have been reached via word of mouth. All NC participants underwent to a comprehensive health questionnaire for screening purposes. Participants were excluded if the following issues were present: history of medical condition, psychiatric illness, substance abuse and/or a neurological disorder.

Demographic information for study participants divided per gender as well as MMSE scores can be found in Table 1 and Table 2. 

The research protocol has been reviewed and approved by the Ethics Committee of the Umberto I University Hospital, Rome, Italy (1055/2021) dated 24 November 2021 in conformity with the principles of the Declaration of Helsinki.

### 2.2. Procedures

Patients were addressed by the referring psychiatrist, and they all received detailed explanation about the aims and characteristics of the study; any additional information requested was provided, further stressing the voluntary and anonymous participation to the study. Subsequently, the informed consent form to the research was presented and signed by each participant. Patients were then assessed by a ward trained psychiatrist by means of the following rating scales: Mini Mental State Examination (MMSE) [16], Brief Psychiatry Rating Scale (BPRS) [17], and finally the Hamilton Depression Rating Scale (HAM-D) [18] and the Hamilton Anxiety Rating Scale (HAM-A) [19].

### 2.3. Clinical and Cognitive Assessment

The 24-item BPRS assessed current psychiatric symptoms severity [16]. The scale comprises 24 items investigating the main psychiatric signs and symptoms. Each item is rated on a 7-point Likert scale ranging from 1 (not present) to 7 (extremely severe) and a total score is defined. 

Cognitive functioning was assessed by means of the MMSE, a screening tool for cognitive functioning; scores equal to or lower than 24 were considered indicative of the presence of cognitive disorders [20].

The HAM-D was employed aiming at assessing patients suffering from affective disorders [17]. The scale contains 24 items with a 5-point Likert scale response. 

The HAM-A is first scales developed to assess the severity of anxiety [18]. It contains 14 items that measure both somatic and psychological anxiety. 

### 2.4. Sexuality Assessment

Sexual aspects were assessed by means of specific questionnaires each of which was employed in two versions according to patient’s gender: the *Brief Index of Sexual Functioning for Women* (BISF-W; Italian adaptation) [21] is a self-assessment tool consisting of 22-questions (64-item) that investigate both qualitative and quantitative aspects of women’s sexual experience. Specifically, it measures female sexual functioning over the past 30 days. The version validated by Panzeri et al. [21] was here employed. In the Italian validation four factors were defined: *Couple sexuality*, *Self-eroticism*, *Sexual discontent*, and *Anal sexuality*. The first factor, *Couple sexuality*, includes desires, frequency, arousal, and orgasm for activities involving the partner; the second factor called *Self-eroticism* includes desires, frequency, arousal, and orgasm for all solipsistic activities (e.g., masturbation). The third factor measures *Sexual discontent* about sexual issues with one’s partner: potential troubles affecting sexual activity such as a woman’s opinion about the reported sexual discontent of her partner are evaluated. The fourth factor *Anal sexuality* includes desires, frequency, arousal, and orgasm achieved in anal intercourse. Higher scores indicate greater sexual functioning. The *Brief Index of Sexual Functioning for Men* (BISF-M; Italian adaptation) [22] is a self-assessment tool consisting of 22 questions (66 items) that provide a quantitative and qualitative assessment of normal male sexual functioning. The questionnaire was constructed by Raoli & Panzeri [22] based on the BISF-W, to directly address psycho-behavioral aspects of male’s sexuality: this questionnaire was conceived to assess men’s sexual functioning avoiding simplified approaches mainly concerning physiological factors such as the degree of penile erection or the ejaculatory capacity of men. The factors that emerged in the questionnaire are the same as those reported in the questionnaire for women, with *couple sexuality*, *self-eroticism*, *sexual discontent*, and *anal sexuality*. Higher scores indicate greater sexual functioning.

The *Sexual Quality of Life-Female* (SQoL-F) [23] is a self-report assessment that measures sexual dysfunctions’ impact on women’s quality of life. The questionnaire was developed by Symonds et al. [23] and is based on Spitzer’s Quality of Life model in which physical, emotional, psychological and social components are included. The questionnaire specifically investigates sexual self-esteem, emotionality, and relationship problems involving sexuality; these factors are measured by 18 items answered on a Likert scale from 1 to 6 defining a total score ranging between 18–108. Higher scores within the scale indicate a better quality of life for women. 

The *Sexual Quality of Life-Male* (SQoL-M) [24] is a self-report instrument developed by Abraham et al. [24] to assess the quality of sexual life in men with sexual dysfunctions: it consists of 11 items with a total score ranging from 11 to 66. Each item has a 6-point Likert-type scale ranging from 1 “completely agree” to 6 “completely disagree”. Higher scores indicate a better quality of life for men [24].

Finally, the *Sexual Dysfunction Questionnaire* (SDQ) [25], a single questionnaire for both genders, was administered to assess the presence of sexual dysfunctions. This questionnaire consists of 19 self-reported items defining a total score of 45 which is the optimal cut-off for differentiating men and women with and without sexual dysfunctions, particularly concerning problems of arousal and sexual desire. 

### 2.5. Statistical Analysis

Statistical analyses were conducted using the Statistical Package for Social Science (SPSS; Version 25.0; IBM SPSS, Armonk, NY, USA). The internal consistency of the employed instruments was tested by Cronbach’s alphas, with an alpha ranging from a minimum of 0.659 to a maximum of 0.924. Descriptive analyses with means e standard deviation were performed. We used an independent sample *t*-test to determine group differences between male NC (MNC) and male PP (MPP), and differences between female NC (FNC) and female PP (FPP). Statistical significance was defined as *p* < 0.05. The distributions of all data were verified for normality. All the statistical analyses were performed on de-identified data.

## 3. Results

### 3.1. Sample Characteristics

Table 1 and Table 2 summarizes sample’s characteristics about differences in men and women. In both the male and female samples there were significant differences between groups in regard to age, education level and occupation. In the male sample there is a significant difference in the marital status variable (χ^2^_3.49_ = 12.47, *p* < 0.002) while this difference is not significant in the female sample (χ^2^_2.49_ = 3.58, *p* = 0.167). As expected, in both the male and female samples, the NC group performed significantly better than the PP group at the MMSE. According to the above-mentioned cutoff scores of SDQ, in the current sample 40 patients with psychosis (86.47%) had sexual dysfunction. Table 3 shows the class of medications taken by patients with psychosis in the sample.

### 3.2. Sexuality Life Comparison for Male Patients with Psychosis and Male NC Groups

Concerning the factor *Couple Sexuality*, a statistically significant result was found (Table 4) with the MNC group showing a higher mean score (M = 74.10, SD = 28.68) compared to the MPP group ((M = 31.55, SD = 32.99), *t* (47) = −4.801, *p* < 0.001, *d* = 1.38). Concerning *Self-eroticism,* the MNC group (M = 63.07, SD = 15.05) showed a significantly higher mean score compared to the MPP group ((M = 30.75, SD = 20.46), *t* (47) = −6.376, *p* < 0.001, *d* = 1.80). Levene’s test indicated unequal variances (F = 9.72, *p* = 0.003); thus, the degrees of freedom were adjusted from 47 to 28. 

In regard of the *Sexual Quality of Life* a statistically significant result was observed (Table 4) and pairwise comparisons showed a higher mean score in the MNC group (M = 5.02, SD = 1.12) compared to the MPP group ((M = 4.06, SD = 1.42), *t* (47) = −2.637, *p* = 0.011, *d* = 0.75). 

For *Sexual Dysfunction* dimension a statistically significant result was observed (Table 4) with pairwise comparisons showing a higher mean score in the MPP group (M = 64.55, SD = 10.74) compared to the MNC group (M = 58.55, SD = 4.22), *t* (47) = 2.278, *p* = < 0.001, *d* = 1.30. 

For *Sexual Discontent* (*t* (47) = −0.348, *p* = 0.704, *d* = 0.10) and *Anal Sexuality* dimensions (*t* (47) = −1.325, *p* = 198, *d* = 0.38) no significant differences were observed between groups. 

### 3.3. Sexuality Life Comparison for Female Patients with Psychosis and Female NC Groups

Concerning *Couple Sexuality* dimension, the FNC group had a significantly higher mean score (M = 75.30, SD = 31.53) compared to the FPP group ((M = 29.62, SD = 26.30), *t* (47) = −5.530, *p* < 0.001, *d* = 1.57 (see Table 5)). In regard of *Self-eroticism* dimension, results showed a significantly higher mean score in the FNC group (M = 16.43, SD = 9.39) compared to the FPP group ((M = 6.12, SD = 6.31), *t* (38) = −4.457, *p* < 0.001, *d* = 1.29). Levene’s test indicated unequal variances (F = 7.07, *p* = 0.011); thus, the degrees of freedom were adjusted from 47 to 38. In regard to *Anal Sexuality* dimension results showed a significantly higher mean score in the FNC group (M = 3.22, SD = 3.33) compared to the FPP group ((M = 0.81, SD = 2.94), *t* (47) = −2.691, *p* = 0.010, *d* = 0.77). 

In regard of the *Sexual Quality of Life* (Table 5), pairwise comparisons showed a significantly higher mean score in the FNC group (M = 4.68, SD = 0.49) compared to the FPP group ((M = 4.11, SD = 1.03), *t* (36) = −2.557, *p* = 0.015, *d* = 0.71). Levene’s test indicated unequal variances (F = 13.01, *p* = 0.001); thus, the degrees of freedom were adjusted from 47 to 36.

Pairwise comparisons concerning *Sexual Dysfunction* dimension (Table 5) showed a significant difference mean in the FPP group (M = 63.24, SD = 11.74) compared to the FNC group (M = 62.34, SD = 4.07), *t* (46) = 0.346, *p* < 0.001, *d* = 0.100). 

*Sexual Discontent* dimension did not show significant differences between groups (*t* (47) = 0.526, *p* = 0.602, *d* = 0.15).

## 4. Discussion

The present study aimed at investigating sexuality and the impact of its dysfunctions on the quality of life of individuals with psychosis compared to a group of healthy controls. Significant differences between psychotic individuals and healthy controls in several areas of their sexual functioning were found: the control group seemed to better perceive *Couple sexuality, Self-eroticism,* and overall appeared to have a higher *Quality of sexual life*; on the other hand, the group of patients with psychosis displayed higher scores in *Sexual dysfunction*. These results equally regarded both men and women and appear in line with previous evidence from the literature [26,27], suggesting that psychosis deeply impacts on patients’ sexuality and on their general functioning as well. Interestingly, no significant differences were found between men with psychosis and healthy men concerning *Anal sexuality* and *Sexual discontent* dimensions. For women, *Sexual discontent* scores did not show significant differences between psychotic patients and healthy controls as well. The *Sexual discontent* dimension refers to specific aspects of the relationship with one’s sexual partner concerning the issues that can negatively influence this relationship. It may be suggested that individuals with psychosis, due to mentalization deficits [28,29], experience difficulties in recognizing their sexual problems or even more in assuming mental illness as a determinant factor for their sexual impairments, thus partially explaining the absence of differences between patients and healthy controls in reporting *Sexual discontent*. On the other hand, regarding the *Quality of life*, psychotic patients showed lower scores compared to healthy controls. These findings are in line with previous studies: MacDonald et al. [30] compared male patients with schizophrenia and community controls observing that patients had higher problems in reporting about sexual desire, in achieving and maintaining an adequate penile erection, displaying early ejaculation and lower rates of satisfaction with orgasm. Esan & Esan [31] reported that person suffering from schizophrenia were unable to achieve orgasm followed by an adequate level of satisfaction. Ghormode et al. [32] described the lack of sexual drive and arousal as the most common sexual problem observed in persons with schizophrenia, at a significantly worse degree than the control group. Previous studies have pointed out how schizophrenia may differently affect the sexual life of men and women, reporting that the latter are more prone to engage in sexually active behaviors [33]. Similar findings have been reported for Autistic Spectrum Disorders (ASD), with affected women appearing more engaged in romantic and sexual experiences compared to men although at higher risk of negative sexual experiences [34].

Regarding the sexual desire as investigated both in *Couple sexuality* and *Self-eroticism* dimensions, patients with psychosis scored significantly lower than the control group. Fan et al. [35] found an altered libido (e.g., the ‘‘biological’’ side of desire) both for men (79%) and for women (65%) in patients with schizophrenia by means of the employment of a subscale for desire. In contrast, Huguelet et al. [36] reported several difficulties regarding both sexual activity and psychological aspects related to sexual functioning (e.g., sexual esteem, motivation, anxiety) for women with schizophrenia compared to age matched healthy controls; however, no significant differences between groups were found in regard of dyadic and individual sexual desire. Moreover, the same study found that only half of recruited women with psychosis resulted to have a sexually active life either in couples or alone. Such findings suggest the absence of significant impairments concerning solitary sexuality in women with psychosis, further indicating that their sexual dysfunctions likely arise from disturbances to intimacy and dyadic relationships. 

Overall, our findings showed that the prevalence of sexual dysfunctions in people with a psychotic disorder was of 86.47%, in line with the figures from European frame equal to 74–96% [37]. It is known that sexual dysfunctions in psychotic disorders involve a complex network of interactions between biological, psychological, and social factors that can be either associated or not to the underlying pathology or its treatment [38]. Indeed, although negative side effects of medical treatments have been accounted for in determining sexual dysfunctions, it has been noticed that psychosis represents a potential cause of such impairments. Studies on un-medicated psychotic patients have found that sexual dysfunctions were present regardless of medication treatment [39,40]. Moreover, the severity of psychotic symptoms was positively correlated with sexual dysfunctions’ burden [41]. Several studies have corroborated the role of psychotic symptoms in the pathophysiology of sexual dysfunctions suggesting that either positive, negative, and cognitive symptoms are involved in difficulties in establishing and maintaining steady relationships [42]. Škodlar & Nagy [12] suggested as a common denominator of sexual dysfunctions in psychotic individuals their specific difficulties in regulating interpersonal closeness: patients seem to be overwhelmed by the presence of other people, feeling even more uncomfortable when interacting with a potential sexual partner. These avoidant behaviors would preclude the opportunity for patients to establish close relationships with others, blaming themselves for their shortcomings to the point of feeling inadequate as potential sexual partners. 

Notably, it has been supposed that several environmental factors contribute to sexual dysfunctions onset in psychotic patients. Moreover, it may be of interest noting that potential changes in cultural and socio-sexual frame of western countries might influence societal assumptions about gender roles and sexual habits [43,44], likely accentuating psychotic patients’ sexual distress. Further studies aimed at deepening the influence of cultural factors on patients’ sexual habits and dysfunctions are required. Complications in maintaining intimate relationships may also arise from partners who would display a prejudicial approach with a decreased sexual desire due to the diagnosis of schizophrenia [9]. The reluctance of psychiatrists and physicians to address this theme with patients adds further difficulties in managing sexual dysfunctions and their burden on patients’ functioning. However, in recent decades both patients and clinicians have appeared increasingly more aware of the impact of sexual dysfunctions on patients’ quality of life, thus avoiding more and more the underestimation of such issue [45].

Importantly, somatic concerns linked to drugs’ side effects have been pointed out as a major factor in decreasing of patients’ compliance to treatments [46,47]. 

## 5. Limitations

The present study has several limitations, among which: (i) our study did not report patients’ pharmacological treatments which likely have influenced results. However, our study design did not seek to define causes of sexual dysfunctions in patients with psychosis but aimed at describing the impact of these impairments in patients’ quality of life; (ii) values and norms related to sexuality and sexual practices are deeply grounded in cultural aspects. In addition, many of the patients with psychosis stated that they were single. The “Couple Sexuality” and “Sexual Discount” scales of the BISF-W and BISF-M consider this condition with special items. For future studies, it would be interesting to investigate dyadic sexuality in patients with psychosis and their partners. 

Hence these results need to be approached as related to the occidental setting in which this study was carried out, considering potential cultural influences on sexuality representations and practice; (iii) we enrolled patients who were able to address a clinical interview: as such, a recruitment bias cannot be ruled out and some of the results may not be applicable to patients with acute illness. However, our sample can be considered as closely representative of acute psychotic states given it has been recruited for a substantial amount in a PICU; (iv) it was not possible to include hormonal variables that have a bearing on sexual functioning such as prolactin. Another study design to investigate the correlations of such biological components with patients’ sexual functioning would be required; (v) on several occasions we found a lack of information regarding episodes of childhood sexual abuse, not allowing us to investigate potential correlations between significant trauma and patients’ sexual functioning. 

## 6. Conclusions

This study describes a poor sexual quality of life among patients with a psychotic disorder compared to healthy controls. According to the literature, about 80% of psychotic patients have at least one sexual problem and the majority would like to talk about it. However, the rate of patients who discuss it with their doctor is about 30% [48]. The most common reasons for the lack of dialogue in people with psychosis are decency, shame, and low interest on the part of the doctor [48]. Sexuality still represents a taboo for patients and physicians, and for this reason it must enter more and more into clinical and research practice. Evidence increasingly shows the need for patients’ sexual functioning assessment, considering this dimension in the definition of suited interventions to provide clinical assistance truly adapted to their daily life. Indeed, considering such dimension during clinical assessments might be useful in structuring a therapeutic alliance and in keeping patients’ compliance. In this regard, it has become increasingly accepted to consider patients’ recovery not only as the cessation of the illness, but also the improvement of their quality of life.

## Figures and Tables

**Table 1:** Demographic characteristics of the male sample (*n* = 49).

Male Sample Characteristics	MNC = 29	MPP = 20	*t/*χ^2^	*p*-Value
Age, mean ± SD (range)	34.10 ± 4.2 (27–45)	41.20 ± 12.3 (23–55)	*t* (22) = 2.49	*p* = 0.021 *
Educational level			χ^2^_3.49_ = 9.47	*p* = 0.009 **
Middle school diploma—*n* (%)	1 (3.4)	3 (15)		
High school diploma—*n* (%)	8 (27.6)	12 (60)		
Bachelor’s degree and postgraduate degree—*n* (%)	20 (69.0)	5 (25)		
Marital Status			χ^2^_3.49_ = 12.47	*p* = 0.002 **
In relationship—*n* (%)	17 (58.6)	2 (10.0)		
Legal Separation/Divorced—*n* (%)	0 (0.9)	1 (5.0)		
Single—*n* (%)	12 (41.4)	17 (85.0)		
Occupation			χ^2^_3.49_ = 6.91	*p* = 0.032 *
Employed—*n* (%)	28 (96.6)	14 (70.0)		
Unemployed– *n* (%)	1 (3.4)	5 (25.0)		
Inable		1 (5.0)		
Alcool use in the last 12 months—*n* (%)	23 (79.3)	9 (45.0)	χ^2^_1.49_ = 6.15	*p* = 0.013 *
Substance use in the last 12 months *—*n* (%)	3 (10.3)	1 (5.0)	χ^2^_1.49_ = 0.451	*p* = 0.502
Tobacco use in the last 12 months—*n* (%)	18 (62.1)	6 (30.1)	χ^2^_1.49_ = 4.87	*p* = 0.027 *
Caffeine use in the last 12 months—*n* (%)	28 (96.6)	20 (100)	χ^2^_1.49_ = 0.704	*p* = 0.401
Mini-Mental State Examination, mean ± SD (range)	29.97 ± 0.19 (29–30)	26.12 ± 2.10 (23–30)	*t* (19) = −6.70	*p* < 0.001 ***

* *p* < 0.05; ** *p* < 0.01; *** *p* < 0.001.

**Table 2:** Demographic characteristics of the female sample (*n* = 49).

Female Sample Characteristics	FNC = 23	FPP = 26	*t*/χ^2^	*p*-Value
Age, mean ± SD (range)	35.68 ± 6.5 (26–49)	46.28 ± 12.3 (20–55)	*t* (39) = 3.95	*p* <0.001 ***
Educational level			χ^2^_2.49_ = 20.26	*p* < 0.001 ***
Middle school diploma—*n* (%)	0 (0.0)	5 (19.2)		
High school diploma—*n* (%)	4 (17.4)	16 (61.5)		
Bachelor’s degree and postgraduate degree—*n* (%)	19 (82.6)	5 (19.2)		
Marital Status			χ^2^_2.49_ = 3.58	*p* = 0.167
In relationship—*n* (%)	14 (60.9)	9 (34.6)		
Legal Separation/Divorced—*n* (%)	1 (4.3)	1 (4.3)		
Single—*n* (%)	8 (34.8)	16 (61.5)		
Occupation			χ^2^_1.49_ = 9.80	*p* = 0.002 **
Employed—*n* (%)	21 (91.3)	13 (50.0)		
Unemployed– *n* (%)	2 (8.87)	13 (50.0)		
Alcool use in the last 12 months—*n* (%)	22 (95.7)	16 (61.5)	χ^2^_1.49_ = 1.58	*p* = 0.208
Substance use in the last 12 months *—*n* (%)	3 (13.0)	0 (0.0)	χ^2^_1.49_ = 4.92	*p* = 0.085
Tobacco use in the last 12 months—*n* (%)	6 (26.1)	10 (38.5)	χ^2^_1.49_ = 2.31	*p* = 0.129
Caffeine use in the last 12 months—*n* (%)	18 (78.3)	26 (100)	χ^2^_2.49_ = 6.29	*p* = 0.043 *
Mini-Mental State Examination, mean ± SD (range)	30 (30)	26.35 ± 2.41 (21–30)	*t* (47) = −2.41	*p* = 0.020 *

* *p* < 0.05; ** *p* < 0.01; *** *p* < 0.001.

**Table 3:** Pharmacological class taken in the sample of patients with psychosis (*n* = 46).

Pharmacological Treatment	Prevalence
Drug Naïve—*n* (%)	3 (6.5)
Antipsychotics—*n* (%)	20 (43.5)
Mood Stabilizers—*n* (%)	13 (28.3)
Antidepressants—*n* (%)	5 (10.9)
Benzodiazepine—*n* (%)	11 (23.9)
Internist drugs—*n* (%)	5 (10.9)
Depot—*n* (%)	2 (4.3)

**Table 4:** Comparison between groups in male sample (*n* = 49; Independent sample *t*-test).

	*t*	df	Sig.	*d*	Multiple Comparisons	Mean Differences	SE
CoSex	−4.801	47	<0.001 ***	1.38	MNC vs. MPP	−42.55	8.86
Self-Er	−6.376	47	<0.001 ***	1.80	MNC vs. MPP	−32.32	5.07
SexDisc	−0.348	28	0.704	0.10	MNC vs. MPP	0.65	1.85
AnSex	−1.325	47	0.198	0.38	MNC vs. MPP	−1.87	1.44
SQoL	−2.524	47	0.011 **	0.75	MNC vs. MPP	−0.96	0.36
SexDys	2.278	47	<0.001 ***	0.79	MNC vs. MPP	6.00	2.20

** *p* < 0.01; *** *p* < 0.001; Abbreviations: CoSex = Couple Sexuality; Self-Er = Self-Eroticism; SexDisc = Sexual Discontent; AnSex = Anal Sex; SQoL = Sexual Quality of Life; SexDys = Sexual Dysfunction; MNC = Male Healthy adult Control; MPP = Male Patients with a Psychotic disorder.

**Table 5:** Comparison between groups in female sample (*n* = 49; Independent sample *t*-test).

	*t*	df	Sig.	*d*	Multiple Comparisons	Mean Differences	SE
CoSex	−5.530	47	<0.001 ***	1.57	FNC vs. FPP	−45.69	8.26
Self-Er	−4.457	38	<0.001 ***	1.29	FNC vs. FPP	−10.32	2.32
SexDisc	0.526	47	0.602	0.15	FNC vs. FPP	0.729	1.39
AnSex	−2.691	47	0.010	0.77	FNC vs. FPP	−2.41	1.44
SQoL	−2.557	36	0.015 **	0.71	FNC vs. FPP	−0.58	0.23
SexDys	0.346	47	<0001 ***	0.10	FNC vs. FPP	0.892	2.58

** *p* < 0.01; *** *p* < 0.001; Abbreviations: CoSex = Couple Sexuality; Self-Er = Self-Eroticism; SexDisc = Sexual Discontent; AnSex = Anal Sex; SQoL = Sexual Quality of Life; SexDys = Sexual Dysfunction; FNC = Female Healthy adult Control; FPP = Female Patients with a Psychotic disorder.

## Data Availability

Data are available under reasonable request to benedetta.barchielli@uniroma1.it.
